# Exploring provider perspectives on respectful maternity care in Kenya: “*Work with what you have”*

**DOI:** 10.1186/s12978-017-0364-8

**Published:** 2017-08-22

**Authors:** Charity Ndwiga, Charlotte E Warren, Julie Ritter, Pooja Sripad, Timothy Abuya

**Affiliations:** 1 Population Council, PO Box 17643-00500, Nairobi, Kenya; 2 Population Council, 4301 Connecticut Ave NW, Suite 280, Washington, DC 20008 USA; 3 St. Jude Children’s Research Hospital, Memphis, TN USA

**Keywords:** Mistreatment, Disrespect and abuse, In-humane treatment, Caring behavior, Respectful maternity care, Behavior change, Interventions

## Abstract

**Background:**

Promoting respect and dignity is a key component of providing quality care during facility-based childbirth and is becoming a critical indicator of maternal health care. Providing quality care requires essential skills and attitudes from healthcare providers, as their role is central to optimizing interventions in maternity settings.

**Methods:**

In 13 facilities in Kenya we conducted a mixed methods, pre-post study design to assess health providers’ perspectives of a multi-component intervention (the Heshima project), which aimed to mitigate aspects of disrespect and abuse during facility-based childbirth. Providers working in maternity units at study facilities were interviewed using a two-part quantitative questionnaire: an interviewer-guided section on knowledge and practice, and a self-administered section focusing on intrinsic value systems and perceptions. Eleven distinct composite scores were created on client rights and care, provider emotional wellbeing, and work environments. Bivariate analyses compared pre- and post-scores. Qualitative in-depth interviews focused on underlying factors that affected provider attitudes and behaviors including the complexities of service delivery, and perceptions of the Heshima interventions.

**Results:**

Composite scales were developed on provider knowledge of client rights (Chronbach α = 0.70), client-centered care (α = 0.80), and HIV care (α = 0.81); providers’ emotional health (α = 0.76) and working relationships (α = 0.88); and provider perceptions of management (α = 0.93), job fairness (α = 0.68), supervision (α = 0.84), promotion (α = 0.83), health systems (α = 0.85), and work environment (α = 0.85). Comparison of baseline and endline individual item scores and composite scores showed that provider knowledge of client rights and practice of a rights-based approach, treatment of clients living with HIV, and client-centered care during labor, delivery, and postnatal periods improved (*p* < 0.001). Changes in emotional health, perceptions of management, job fairness, supervision, and promotion seen in composite scores did not directly align with changes in item-specific responses. Qualitative data reveal health system challenges limit the translation of providers’ positive attitudes and behaviors into implementation of a rights-based approach to maternity care.

**Conclusion:**

Behavior change interventions, central to promoting respectful care, are feasible to implement, as seen in the Heshima experience, but require sustained interaction with health systems where providers practice. Provider emotional health has the potential to drive (mis)treatment and affect women’s care.

**Electronic supplementary material:**

The online version of this article (doi:10.1186/s12978-017-0364-8) contains supplementary material, which is available to authorized users.

## Plain english summary

Many women encounter uncaring and abusive treatment from health care providers during facility-based labor and delivery. In this study, the *Heshima* project sought health care providers’ opinions and experiences in implementing a package of activities designed to promote caring and humane treatment during facility-based childbirth.

Health providers working in maternity units were interviewed on their knowledge and practice of women’s childbirth rights as well as how their working conditions, supported or limited their ability to provide caring behaviors. Providers were also given a self-administered questionnaire focusing on their own attitudes, behavior, supervisors, and health care systems.

Eighty-nine and thirty-three providers were interviewed at baseline and endline, respectively. There was a significant increase in the proportion of providers identifying elements that constitute clients’ rights: ‘explaining to clients when and how procedures will be performed and the outcomes’, from 38.8% to 63.2% at endline. High case loads, work-related stress, and un-supportive work environments limited providers’ efforts in offering dignified and respectful care.

In conclusion, our results show that interventions promoting providers’ positive behaviors and attitudes are feasible to implement but require a sustained, supportive health system environment. Provider working relationships and environments not only affect their own emotional health, but also how they treat mothers during childbirth.

## Background

Disrespect and abuse of pregnant women seeking maternity services persists in high, middle, and low income settings [1–9]. For decades, ‘unfriendly’ and ‘poor’ attitudes from health care providers have been described, by women, as barriers to maternal care [10, 11]. Weak health systems contribute to this poor access and use of maternal health services by amplifying the negative effects of disrespect and abuse during childbirth. Existing quality of care frameworks focus on reducing adverse maternal and newborn outcomes, and emphasize caring for women with respect and dignity as an integral part of reducing mistreatment during childbirth [11–13].

The current discourse in promoting respectful maternity care (RMC) is driven by several initiatives attempting to address women’s mistreatment during labor and delivery, including the Humanizing Childbirth Movement [14], the Better Births Initiative [15], and more recently, studies implemented between 2011 and 2015 in Ethiopia, Kenya (the *Heshima* Project), Ghana, Nigeria, and Tanzania [4, 5, 7, 16, 17]. These recent studies built upon findings from a 2010 landscape review that reiterated knowledge and practice gaps in women’s treatment during this vulnerable period [1]. Attempts to better define disrespect and abuse during childbirth [18], as well as describe and measure its occurrence, have contributed to the development of a range of evidence-based interventions [7, 19, 20]. Strategies for resolving this problem increasingly use a rights-based approach to ensure health providers’ and their managers’ accountability [11].

Health providers’ role in ensuring safe delivery and promoting respectful maternity care in unsupportive and weak health systems environments often conflicts with accepted professional norms, increasing susceptibility to propagate mistreatment. Evidence shows that drivers of mistreatment in facilities are multidimensional, ranging from individual providers to organizational and systemic factors [1, 21]. Facility influences on efforts to reduce women’s mistreatment include staff attitudes, motivation, and maternity care service governance [19]. With increasing recognition of these social and organizational factors, provider perceptions and responses for promoting RMC initiatives remain part of the core to their success. It is therefore critical to study and assess providers’ understanding and experience in promoting RMC [22]. This paper describes providers’ perceptions, attitudes, knowledge, and practices for both their work environment and clients’ rights after a behavior change intervention package aimed at promoting RMC in Kenya.

### Overview of intervention tested at facility level

The *Heshima* project in Kenya (June 2011–February 2014) used a participatory process for developing and implementing a package of interventions at facility, policy, and community levels [19, 23, 24]. Health facility interventions included health care providers and their managers to improve providers’ attitudes, working environments, facility management, and links to communities. The principal approach supported managers’ and health providers’ critical self-evaluation and modification of their values, attitudes and beliefs. One- and three-day workshops for 132 health managers and 146 providers, respectively, utilized materials adapted from the values clarification and attitude transformation (VCAT) curricula developed by IPAS [25]. VCAT workshops built providers’ capacities to recognize disrespect and abuse and improved their knowledge and application of international and national laws and conventions including treaties on reproductive health and human rights, as well as their professional ethics, facility accountability, and management. The workshops emphasized provider and client rights and obligations during childbirth. *Heshima* also worked with facilities’ quality improvement teams (QITs) to streamline management of their resources, such as ensuring appropriate maternity staff allocation and essential maternity commodities and drugs. Counseling or psychosocial support (in group or individual sessions) enabled providers to discuss work challenges and related pressures. Maternity Open Days, where maternity staff invited pregnant women and their families to tour labor wards, provided opportunities for women to learn about labor and delivery procedures, demystifying the birth process, along with discussing birth planning with male partners and other family members.

These interventions were implemented during a series of political and policy changes starting with Kenya’s new constitution in 2010, which devolved resources and power from the national government to its 47 counties in effect after the March 2013 general election [26]. Counties assumed greater health system roles and responsibilities including financing, governance, human resources, procurement, and logistics [27]. A second policy influence on this study’s implementation was the introduction of free maternity care in public health facilities in 2013. Increased workloads following these changes and uncertainty in salary sources (e.g. national or county) resulted in two nursing strikes of 2 to 3 months affecting service delivery and slowing implementation in study facilities. Table 1 shows the participants reached, duration, periodicity, and indicators associated with each intervention.

**Table 1:** Provider/Facility level interventions

Goal: To promote Respectful Maternity Care						
Expected out comes; Reduce incidents of D & A; improve provers attitude and work environment						
	Intervention areas	Priority focus on forms of D&A	Proportion or number of participants reached^a^	Duration	Periodicity during the intervention	Indicator/measurements *(baseline and endline results)*
b.	*Training of providers on strategies that improve provider attitude* -Training of providers using value clarifications and attitude transformation change approach (VCAT)	All forms of D&A	90% (*n* = 132) managers trained on RMC across all project sites 62% (*n* = 146) providers trained in project 13 sites ranging from 7 to 45 of providers in maternity units	2 days 3 days	One provider and one manager workshop per county Quarterly - Two to three on site mentorship sessions per sites	*-% of providers who report improved attitude as a result of the intervention*
*c.*	*Psycho-social support for health care providers* -Providing groups and individual counselling session for providers -establish psycho-social support structures between facilities and communities	All forms of D&A	49% (*n* = 113) providers (8–12 per site) 77% (*n* = 10) *facilities with functional referral mechanisms for psycho-social support structures in project sites*	45 min- 1 h	Quarterly	*-% of facilities that have continuous counselling sessions for providers*
*d*.	*Maternity Open Days*	All forms of D&A	100–300 persons (depends on facility size and location)	24 (total) for 1 day All study sites conducted at least one session Ranged from 1 to 4 session per facility and reached	Quarterly	-% of facilities that conduct maternity open days at least once every quarter - % of provider reporting improved client provider interaction -% of clients who have correct knowledge on birthing process and procedures
*e*	*Establishment of multi-disciplinary peer support groups/ watch dogs*	All forms of D&A	53% (*n* = 13) of facilities with functional peer support groups in facility catchment sites -Instances where any cases of D & A are discussed and amicably resolved	7 out of 13 facilities established multi-disciplinary peer support groups/watch dogs	Quarterly	-% of D&A cases reported and amicably resolved

^a^ *denominators vary from one intervention to the other*

## Methods

### Study design

In order to assess the effects of the Heshima project at provider level we conducted a pre-post study, without a comparison group, to describe provider attitudes before and after interventions to reduce disrespect and abuse during childbirth in 13 Kenyan health facilities in five counties (including Nairobi) in Central and Western Kenya [19, 23]. Researchers collected baseline data between September 2011 and February 2012, and endline data between January and February 2014. The 13 purposefully selected facilities comprised different facility types (public, private, faith-based) and levels of care, with three referral hospitals, three district hospitals with maternity units, two faith-based hospitals, two maternity homes, and one health center. Four facilities were rural, and the rest were in urban or peri-urban areas.

### Data sources and collection

This paper is based on two sets of data collected.Quantitative interviews with health care providers (nurse-midwives) and qualitative in-depth interviews (IDIs) with health care providers (nurse-midwives, doctors), facility-in-charges (nurse-midwives) and facility managers, senior reproductive health program managers (at national, county, and sub-county levels), and civil society representatives. Eighty-nine IDIs (N_baseline_ = 56; N_endline_ = 33) were conducted with purposively selected providers with at least 6 months’ work experience. Interviews were conducted at times of providers’ convenience at their place of work. At each site two or three providers were interviewed on their knowledge and perceptions of women’s childbearing rights, their own attitudes and behaviors toward clients, their work environments and related stress, and experiences with RMC interventions. IDIs sought additional information on systemic and governance factors that could have contributed to abuse and disrespect. Whenever possible, the same providers were interviewed at baseline and endline; however, due to the frequency of transfers within the health system and challenges due to the devolution process, only 20% of providers interviewed at baseline, and attended the primary VCAT workshops, were available to be interviewed again at endline.

A total of 142 quantitative interviews with providers (N_baseline_ = 67; N_endline_ = 75) were conducted in the 13 facilities. All providers responsible for maternal health services in a facility available on the day(s) of data collection were interviewed (Table 2). Research assistants conducted the first part of the quantitative questionnaire, while the second half was self-administered. Both components featured questions on provider knowledge, attitudes, practice, and experiences working in maternity units. Demographic characteristics, membership in professional organizations, and salary information was also collected.

**Table 2:** Data collection methods and type of study participants

Baseline				End line	
Category of participants	Methods	Type of respondents	Number of participants	Type of respondents	Number of participants
Policy makers	In depth Interviews	Policy makers in health and program civil society leaders, health rights advocates at national and county level	23	County Health managers	10
Health providers	In depth Interviews	Facility managers, Maternity ward or unit in charges	56	Facility managers, Maternity ward or unit in charges	23
	Quantitative Structured interviews	49 first line services providers in maternity units and 18 managers interviewed for IDIs	67	69 first line services providers in maternity units and 6 managers interviewed for IDIs	75

The second part of the study tool, originally developed and validated in South Africa [28], used a Likert type scale focusing on providers’ perceptions of working conditions, respect, client empathy and prejudice, awareness of policy and service delivery guidelines on respect, dignity and client rights, along with questions on staff turnover, absenteeism, vacancy rates, workload, motivation, with challenges in managing and retaining maternity staff also included.

### Data management and analysis

#### Qualitative data analysis

**Fig. 1:**
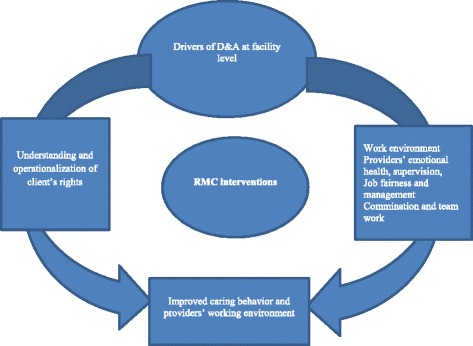
Relation between D & A drivers and provider’s perspective on RMC interventions

Qualitative IDIs were recorded, transcribed verbatim, and translated into English (where necessary). Transcripts were coded using QRS Nvivo Software Version 10 and thematically analyzed using a combined approach with both inductive and deductive coding. Two independent researchers read the transcripts, generated codes, and applied them to baseline and endline data. Inductive codes emerged from the data, while deductive codes were based on existing literature and intervention domains [29, 30]. Codes were then clustered into broader thematic categories. Comparisons were made between baseline and endline data as well as between provider and manager perspectives for insight on the meaning and relationships of themes generated. The research team discussed findings throughout the analytic process, for both reflexivity and rigor. Results were subsequently organized around the centrality of provider attitude and behavior in promoting RMC, understanding and operationalizing client rights, and work environment (Fig. 1).

#### Quantitative data analysis

The quantitative survey directly assessed demographic characteristics, knowledge, professional organization membership, and salary information. Other outcomes related to facility factors, such as provider knowledge and attitudes – many of which are latent constructs that encompass multiple indicators – were developed as composite scores consisting of individual items within the survey. Eleven distinct composite scores were created to assess overall provider attitudes about client treatment (‘client rights’, ‘client-centered care’, ‘HIV care’), providers’ emotional wellbeing, and work environment (management, job fairness, supervision, promotion, system issues). Table 3 describes the scales, individual items comprising the composite scores, and associated reliability using Chronbach’s alpha. The full list of individual items and frequencies are presented in Additional file 1 Table A. These items emerged from prior literature as well as *Heshima*’s formative phase and thus exhibit face and content validity [31].

**Table 3:** Composite measure item list

Measure	Items
Client rights (0–14 items)	Informing/orienting the clients of her where about in the facility/ ward/unit; Explaining clients when and how procedures will be performed and the outcomes; Obtaining consent for all procedures to be done; Informing client of danger signs during labour and delivery and after birth; Informing her of the labour progress and expected possible duration of labour; Allowing her to choose her birthing position; Allowing her to choose a birth partner/companion during labour and delivery; Respecting her privacy; Keeping her information confidential; Ensuring privacy and confidentiality at all time while attending to client during labour and delivery; Respecting her as an individual with her own rights despite her background; Ensuring that the procedures are promptly done within the required guidelines; Ensuring that the mothers labour is monitored using a partograph; Taking client to theatre for caesarean section when is not really necessary.
Client-centered care (0–7 items)	I feel that it is always necessary to obtain consent from clients when conducting a vaginal examination; If a woman’s uterus rupture during labour and delivery and it becomes necessary for the doctor to perform hysterectomy in order to save her life - It is always necessary to inform her of this unplanned procedure before she is discharged home; On admission mothers should be allowed to select the provider/s of their choice during labour and delivery; Mothers who are unable to pay for maternity services should not be detained in the facility to avoid losing the much needed revenue; Treating mothers with care and respect during child birth does make clients come back to this facility; During labor and delivery not being harsh to the mothers ensures that they cooperated with you during procedures; Sharing of beds in this facility is generally not acceptable to the mothers during labour and delivery.
HIV Care (0–7 items)	I do not try to avoid conducting vaginal exam for HIV positive clients; I am comfortable conducting a delivery for a client who is HIV positive; I am comfortable repairing a tear or an episiotomy for a HIV positive client; I do not feel that during labour and delivery the HIV positive client should be isolated from the rest; I am comfortable caring for mother who has HIV soon after delivery; I am comfortable nursing babies born of mother who are HIV positive; Recording clients positive HIV status on the clients card/mother baby booklet make some of the clients uncomfortable.
Emotional health (0–22 items)	Not emotionally drained from my work; Not used up at the end of the workday; Not fatigued or tired when they get up in the morning and have to face another day on the job; Easily understand how their patients feel about things; Do not treat any patients as if they were impersonal objects; Working with people all day is not really a strain for me; Deal very effectively with the problems of their patients; Not burned-out from my work; Positively influence other people’s lives; Have not become more callous/hardened toward people since I took this job; The job is not hardening them emotionally; Very energetic; Not frustrated by my job; I do not feel I’m working too hard on my job; Really care what happens to all patients; Working directly with people does not put too much stress on me; Easily create a relaxed atmosphere with their patients; Accomplish many worthwhile things in this job; Exhilarated after working closely with their patients; Do not feel like they are at the end of my rope; Deal with emotional problems very calmly; Patients do not blame them for any of their problems.
Management (0–14 items)	Job decisions are made by my manager in an unbiased manner; My manager makes sure that all staff concerns are heard before job decisions are made; To make job decisions, my manager collects accurate and complete information; My manager clarifies decisions and provides additional information when requested by staff; All job decisions are applied consistently across all affected staff; Staff are allowed to challenge or appeal job decisions made by my manager; My manager offers adequate justification for decisions made about my job; When making decisions …my manager treats me with kindness and consideration, …my manager treats me with respect and dignity, …my manager is sensitive to my personal needs, …my manager deals with me in a truthful manner, …my manager shows concern for my rights as an employee, …my manager discusses implications of the decisions with me, …my manager offers explanations that make sense to me.
Job fairness (0–5 items)	My work schedule is fair; I think that my level of pay is fair; I consider my workload to be quite fair; Overall the rewards I receive here are quite fair; I feel that my job responsibilities are fair;
Supervision (0–5 items)	I think this is a fair supervision system; I feel good about this supervision system; I am satisfied with this supervision system; The feedback I receive is fair; I think my supervisors are knowledgeable for effective supervision
Promotion (0–5 items)	I think this is a fair promotion system; I feel good about this promotion system; I am satisfied with this promotion system; The promotion opportunities I have are fair; Compared to other people doing similar work, my opportunities for promotion are fair.
Health system (0–9 items)	I think this is a fair system; I feel good about this system; I am satisfied with this system; This system provides fair training opportunities; The outcome of this system is that I get the training I deserve; I am adequately trained for the tasks I perform; Most of the training I have received has improved or changed how I practice; The training I have received in general has been of high quality; The facility management has offered me an opportunity to practice post in-service training.
Work environment (0–13 items)	Enough staff to provide quality patient care; Enough staff to get the work done; Opportunity to work on a highly specialized patient care unit; Adequate support services allow health workers to spend time with patients; Freedom to make important patient care and work decisions; Patient care assignments that support continuity of care, i.e., the same health workers care for the patient from one day to next; Health professionals control their own practice; Adequate pre-service education for my current position; Adequate clinical practical opportunities during pre-service training; Adequate opportunities for professional development and career; Adequate clinical supervision in this service; Consistent availability of supplies and medications to perform my duties; Functioning equipment and infrastructure to perform my duties.
Working relationships (0–14 items)	Enough time and opportunity to discuss patient care problems with other staff; A manager who is provides support supervision and leadership; A manager who backs up the staff in decision-making and conflict resolution even if the conflict is within cadre, below or with a more qualified member of staff; Hospital/clinic managers support and value health workers; Doctors, nurses and other health workers have good working relationships; Medical Officers have good working relationships with Clinical Officers; Nurses have good working relationships with Clinical Officers; Medical officers have good working relationships with Nurse midwives; Obstetricians have good working relationship with midwives; Enrolled nurses have good relationships with registered nurses; Nurses have good relationships with Medical Officers/interns; Nurses have good relationships with doctors; Collaboration (joint practice) between different cadres of health workers; A lot of team work between different cadres of health workers.

For all scales created to assess provider knowledge and attitudes, individual items were dichotomized with 1 representing endorsement of the item or agreement and 0 representing non-endorsement or disagreement with the item. A composite score was then created by summing all the individual items within each scale. Individual items for ‘Client-Centered Care’, ‘HIV Care’, and the five scales addressing work systems (management, job fairness, supervision, promotion, and health system) were originally assessed on a five-point Likert scale and then dichotomized as *strongly agree/agree* versus all other responses. Two additional scales assessing work environment and relationships consisted of items originally assessed on a four-point Likert scale (strongly agree, somewhat agree, somewhat disagree, strongly disagree) were dichotomized as ‘Agree’ versus ‘Disagree’.

Descriptive statistics were provided to characterize the sample at baseline and endline, both for provider characteristics (e.g. age, gender, etc.) and facility or job characteristics (e.g. facility type, current working station of provider). Bivariate analyses using chi-square or t-tests, to determine if baseline and endline participants were significantly different in socio-demographic characteristics or in provider knowledge or attitudes, were assessed at the *p* = 0.05 significance level. Subsequent exploratory bivariate analyses (Chi-square or Fisher’s Exact test in event of small cell sizes) examined differences between baseline and endline for the individual components of each scale to consider what specific components, if any, were driving significant change between the two groups. All quantitative analyses employed SAS software, Version 9.4 (Cary, North Carolina, USA).

## Results

### Characteristics of providers

Providers interviewed during baseline and endline were similar in terms of socio-demographic characteristics, work experience, professional support, and compensation (Table 4)*.* Providers were, on average, 35 years old and had worked in the health sector for 11 years. There was an increase (0% to 38%) in the number of providers serving all areas (admission, antenatal, postnatal, labor, delivery, nursery) at endline, suggesting improved provider functioning through diversified ability to respond as needed across units, although it may also reflect staffing challenges following the influx of patients due to free maternity services.

**Table 4:** Characteristics of health care providers participating in baseline (2012) or endline (2014) surveys of the Heshima project in 13 facilities in Kenya, *N* = 142

Characteristics	Baseline (*n* = 67) % (n)	End line (*n* = 75) % (n)	*p* – value
Facility type			
Hospital	91.0 (61)	90.0 (63)	0.920
Health center	6.0 (4)	5.7 (4)	
Maternity home	3.0 (2)	4.3 (3)	
Type of sector			
Government/council	76.1 (51)	77.1 (54)	0.888
Private or faith based	23.9 (16)	22.9 (16)	
Gender of provider			
Female	82.1 (55)	77.5 (55)	0.500
Male	17.9 (12)	22.5 (16)	
Current working station of provider interviewed			
Admission room	1.6 (1)	10.3 (7)	<.0001
Antenatal room/ward	15.9 (10)	17.7 (12)	
Post-natal ward	14.3 (9)	16.2 (11)	
Nursery	0 (0)	2.9 (2)	
Serving in all areas	0 (0)	38.2 (26)	
Labour/Maternity ward	58.7 (37)	14.7 (10)	
Other	7.9 (5)	0 (0)	
Background			
Doctor/Clinical Officer/Med Intern	13.4 (9)	6.7 (5)	0.211
Nurse/Midwife	86.6 (58)	85.3 (64)	

### Understanding and operationalization of client’s rights

The quantitative and qualitative data supported, by varying degrees, the understanding and operationalization of client rights, an integral component of provider promotion of RMC. Quantitatively, providers’ perceptions of how they treated clients reveal, on average, an increase in their awareness of ‘client rights’ and improvements in ‘HIV care’ and ‘client-centered care’. Providers’ awareness of ‘client rights’ (max score = 14) increased from a mean score of 4.5 (SD = 2.4) at baseline to 6.2 (SD = 3.1) at endline (*p* = 0.001). The mean score for how providers treat women living with HIV (max score = 7) improved from 1.5 (SD = 1.5) at baseline to 4.8 (SD = 1.9) at endline (*p* < .0001). The mean score on selected aspects of ‘client-centered care’ during labor, delivery, and the postnatal period (max score = 7) showed significant improvement, from 1.8 (SD = 1.4) at baseline to 5.6 (SD = 1.3) at endline, (*p* < .0001) (Table 5).

**Table 5:** Composite scores assessing provider knowledge and attitudes on client’s rights among health care providers participating in baseline (2012) or endline (2014) surveys of the Heshima project in 13 facilities in Kenya, *N* = 142

	Baseline (*n* = 67) Mean score (SD)	End line (*n* = 75) Mean score (SD)	*p* - value	Cronbach’s alpha
Clients Rights (0–14)	4.5 (2.4)	6.2 (3.1)	0.001	0.70
Emotional Health (0–22)	16.3 (3.4)	14.8 (3.6)	0.036	0.76
Client Centred Care (0–7)	1.8 (1.4)	5.6 (1.3)	<.0001	0.80
HIV care (0–7)	1.5 (1.5)	4.8 (1.9)	<.0001	0.81

Exploratory analyses of individual items within each scale show how specific components drive these improvements (detail in Additional file 1 Table A). There was a significant increase in the proportion of providers identifying certain aspects of client rights: ‘explaining to clients when and how procedures will be performed and the outcomes’ (38.8% to 63.2%; *p* = 0.005), ‘respecting her privacy’ (62.7% to 81.2%; *p* = 0.016), and ‘respecting her as an individual with her own rights despite her background’ (25.4% to 44.9%; *p* = 0.017). All individual elements showed significantly increased proportions of providers who felt comfortable providing ‘HIV care’ for women living with HIV and their babies. Within the ‘client-centered care’ scale, there was an increase in the proportion of providers endorsing the need for client consent for vaginal examination (18.8% to 86.4%; *p* < 0.0001), along with ensuring cooperation with procedures by not being harsh with women during labor and delivery (25.4% to 89.8%; *p* < .0001).

Qualitative data suggest providers’ made efforts to improve clients’ rights by informing and including clients in the decision-making process during care. It appears there was need for providers’ training on clients’ rights, while some improvements were observed at endline.

“*As we think how to reduce D&A, at the very least* t*rain providers on the clients’ rights and obligations”* (Manager, Baseline)

*“You know in our set up the client is always right so if they say I don’t want to be in this position or I do not want to stand here for long then you explain to the client the risks and benefits then the client makes the decision. ”* (Maternity in-charge, endline)

Challenges remain, however, to improving clients’ rights. Despite facility improvements such as provision of curtains for privacy, respondents felt that staff shortages still hindered implementation of client rights protocols.

“*Because we are short in staffing, we want a quick eye contact both sides of the ward….when you are running to attend to the other cases there, I can be able to spot this mother in bed five very fast … the staffing does not allow us to have good ample one-on-one personalized care … I would not want them to be under the curtain because if she is changing conditions there while am busy doing other things, by the time I realize, things are worse.”* (Midwife, endline)

#### Providers’ work related environment

Work environments played a significant role in shaping providers’ abilities to promote RMC in their work (Fig. 1), specifically affecting provider’s emotional health and perceptions on supervision, job fairness and management, as well as communication and teamwork.

### Emotional health

Overall, the majority (>75%) of providers at baseline and endline indicated good working relationships between different provider cadres. (Table 5)*.* However, providers still experience burnout and emotional strain. A significant decrease (*p* = 0.036) was seen in the composite mean emotional health score (max score = 22) between baseline (16.3, SD = 3.4) and endline (14.8, SD = 3.6). Exploratory analyses of individual emotional health items showed no difference, except a few significant elements of work-related emotional stress (Table 6). More providers felt at the ‘end of their tether’ at endline than at baseline (26% v. 12%, *p* = 0.043), indicating increased levels of frustration and fatigue, may, in part, result from the free maternity mandate that coincided with the later *Heshima* implementation stages.

**Table 6:** Individual composite score items assessing knowledge and attitudes on clients rights of providers participating in baseline (2012) and endline (2014) surveys of the Heshima project in 13 facilities in Kenya, *N* = 142

	Baseline (*n* = 67) % (n)	End line (*n* = 75) % (n)	*p* – value
Client Rights (14 items)			
Informing/orienting the clients of her where about in the facility/ward/unit	41.8 (28)	56.5 (39)	0.086
Explaining clients when and how procedures will be performed and the out comes	38.8 (26)	63.2 (43)	0.005
Obtaining consent for all procedures to be done	50.8 (34)	65.2 (45)	0.087
Informing client of danger signs during labour and delivery and after birth	29.9 (20)	24.6 (17)	0.495
Informing her of the labour progress and expected possible duration of labour	32.8 (22)	43.5 (30)	0.202
Allowing her to choose her birthing position	27.3 (18)	18.8 (13)	0.244
Allowing her to choose a birth partner/companion during labour and delivery	17.9 (12)	24.6 (17)	0.338
Respecting her privacy	62.7 (42)	81.2 (56)	0.016
Keeping her information confidential	37.3 (25)	65.2 (45)	0.001
Ensuring privacy and confidentiality at all time while attending to client during labour and delivery	40.3 (27)	52.2 (36)	0.165
Respecting her as an individual with her own rights despite her background	25.4 (17)	44.9 (31)	0.017
Ensuring that the procedures are promptly done within the required guidelines	29.9(20)	37.7 (26)	0.335
Ensuring that the mothers labour is monitored using a partograph	25.4 (17)	37.7 (26)	0.123
Taking client to theatre for caesarean section when is not really necessary	3.0 (2)	7.3 (5)	0.261

Qualitative findings support providers’ work-related stress, at both baseline and endline.

“…*In the same place... they are very active (referring to midwives)...you can find somebody who has worked overnight and yet is still in the mood of working. That one we can give a locum (extra work for pay) and maybe there is one who cannot handle the work after such a shift.... she is so stressed... but I can you tell both are tired anyway but we do not have enough staffs……we need someone to cover a shift”* (Maternity Manager. baseline).

At endline one respondent said;

*“…Sometimes nurses experience burnout related to work and workload and sometimes related to outcomes like a maternal death. And it would put them in a situation where they are not able to cope*” (Midwife, Endline)

### Perceptions on supervision, job fairness and management

Providers’ perceptions of their supervision, job fairness, management, health facilities, and systems effects are shown in Table 6. Providers felt improvements in supervision as shown by a significant mean score (max score = 5) increase of supervision positive attributes from 1.1 (1.5) at baseline to 3.0 (1.9) at endline (*p* < .0001). They also reported a higher management score (max score = 14) at endline of 7.5 (5.2) than baseline, of 4.2 (4.4) (*p* < 0.0004). By contrast, providers indicated significant decreases on their job fairness scale scores (max score = 5), from 2.9 (1.7) at baseline to only 1.7 (1.4) at endline (p = <.0001).

The decrease in job fairness assessments are likely related to burnout and declines in work-related emotional health *(*Table 6)*.* Between baseline and endline, significantly fewer providers agreed that their pay was fair (69.4% to 11.3%; *p* < .0001), their workload was fair (68.3% to 21.0%; *p* < .0001), and their rewards are fair (77.4% to 16.1%; *p* < .0001). Regardless of the composite score on provider dissatisfaction with workload and compensation, significant increases, from 20% at baseline to over 50% at endline, were seen for all individual indicators of supervision and fairness. Exploratory results demonstrate supervisory improvement: at baseline, only 18% of providers reported manager clarification of decisions and additional information when requested by staff; by endline, this proportion increased to 53.2% (*p* < .0001).

These results triangulate with qualitative results. Respondents’ perceive that high caseloads and limited staff in maternity care resulting in poor care, overworked providers, work-related stress, combined with limited infrastructure for service delivery, negatively influence their attitudes towards their work. Similar facility factors persisted, at both baseline and endline, as challenges to providers’ abilities to promote RMC.

*“Women expect too much from the providers, if the provider does not have curtains or blankets, they can do nothing to ensure comfort and privacy”* (Midwife, baseline).

*“So even if she would appreciate the value and clarification exercise, she is not able to deliver fully to the client as she would also wish to do … the human resource factor is a problem. The other handicap was the space, the infrastructure in itself*.” (Medical doctor, endline).

Qualitative data further show improvements in provider perceptions of manager-provider interactions. Stakeholders as well as baseline results suggest need for an intervention focusing on managers’ leadership roles and responsibility for improving maternity units’ working conditions.

“*Some maternity managers can be trained in leadership and the management should select competent managers in those positions, focus should also be on facility in charges themselves… provide equipment also”* (Facility Manager, baseline).

Managers at endline were described as supportive of both clients and providers engaging in RMC-promoting behaviors. A nurse manager described her role in sustaining *Heshima* practices.

*“We are adhering to what we learnt about respecting our clients…. we are encouraging men to come for ANC where we encourage mothers to come with their spouses… we want them to feel comfortable; we give them a visit round the facility so that in the event they come [for delivery] they know where they will be attended and by who. We are also encouraging the staff to be courteous to the clients…to introduce themselves …make sure they have their name tags properly displayed…”* (Facility manager, endline).

Results also show improved provider management, communication, and teamwork, resulting in better relations between providers and clients.

*“The element of communication really improved; midwives could communicate very well with clients and it reduced some of clients’ perceptions of disrespect and abuse … another thing which worked very well was teamwork, which started at the site of implementation; the maternity itself, the nursing office... right to the Health Management Team.”* (Nurse-midwife & mentorship coordinator, endline)

However, managers – and providers who experience heavy workloads – express frustration with the effort required to ensure clients receive quality and respectful care. One manager lamented about her inability to address understaffing, which may contribute to client rights’ violations.

*“I make the best noise I can to my in-charge to improve staffing in maternity…. And she also raises her hands up (implying giving up) that I have also made noise up there (implying to staffing office) and there have not posted for me staff…. So you will work with what you have. But I will not keep quiet but it is the reality. You want to give the quality care… but it needs the manpower to give it”*. (Reproductive health coordinator, endline)

Providers also reported that the promotion system is unfair, pay inadequate, and demotivating. Provider demotivation across Kenya may have stemmed from three nationwide providers’ strikes that lasted five out of the 20 months of the study’s implementation period, for reasons that included delayed salaries, poor pay, lack of promotion, and poor working conditions. Some providers suggest rewarding good behavior and attitudes to motivate providers offering maternity care services. “*They [providers] should be motivated in a way, even if it’s not allowances or cash but the management or professional should appreciate the unique work that is being carried out at the maternity… we can rate the health provider in the maternity who is doing well … maybe the best appreciated midwife can be recognized and given trophy, something like that.”* (Facility in-charge, endline).

## Discussion

Our study used mixed methods and developed composite measures for individuals and teams to measure behavior, beliefs and attitudes, professional ethics, and self-awareness. The data describes the complexity of factors affecting provider behavior and the challenges they face in the provision of maternal health care. Quantitative data show that more than half the providers reported that they were emotionally drained from their work or exhausted at the end of the workday both at baseline and endline, yet there were improvements in their performance in respecting client rights.

Study findings demonstrate some improvements in behavior, beliefs, and attitudes among providers in maternity units after *Heshima*’s implementation, but challenges remain. Composite scores indicate increased knowledge and improved practices among providers for understanding client rights, client-centered care, and positive attitudes towards HIV-positive clients. Intervention effects on work environments indicate varying levels of improvement. Management (particularly shared decision-making) and supportive supervision increased, while providers reported a reduced sense of job fairness. Provider perspectives of *Heshima*’s implementation are, in part, confounded by the devolution of health governance from national to county levels. Although well intended, devolution created under-resourced work environments (strained leadership and governance capacity) and may have contributed to inadequate management affecting client-provider interactions. We observed varied intervention effects on providers’ emotional health and client-provider relationships.

Our findings demonstrate that positively influencing providers’ understanding of client rights is feasible, but optimizing their attitudes and behaviors for successful implementation of a rights-based approach upholding clients’ rights is complex. Despite the increase in composite scores for clients’ rights and supported qualitative findings, closer investigation of individual measures shows little change over the intervention period. In few cases, even when providers internalized their VCAT training, were they unable to apply due to peer influences, supporting the theory that individual behaviors in health service delivery systems may be affected by ‘groupthink’ or a team’s cultural normative practice [32]. A provider’s ability to critically self-regulate, along with group composition and work dynamics, influence provision of client-centered care and highlights the need to respond to both midwife and client needs [12, 33–35].

Providers’ abilities to deliver women-centered care are moderated by work environment [36, 37]. Our study illustrates improvements in nurses’ experiences with enhanced team efforts and better supervision including a fair review system. Other studies show that conducive work environments foster uptake of the effective midwifery practices, while inter-professional rivalries deter quality of care [38, 39]. Our findings show difficulties in providing client-centered care for various reasons including inadequate space, low staffing, and high client loads, leading to provider burn-out particularly in in big facilities with higher volume maternity units. The need for adequate staff with a positive attitude for maternity care, along with adequate space, equipment, supplies, and commodities is integral to women-centered care [19, 36, 40].

This study elevates the necessity of providers’ emotional well-being to deliver respectful and dignified care in overwhelmed work environments. Nurses’ and junior providers’ psychological health are heavily influenced by how supportive management styles and communication structures are [41]. Despite the high acceptability of counseling among providers and managers, its effect on emotional health was not statistically significant, likely because of limited session frequencies and variability in type and quality of identified counselors. Counselors described as ‘too familiar’ with nurses in maternity units often were less effective in providing psychosocial support.

Minimal improvements in work environment and working relationships between baseline and endline affected staff emotional well-being. Poor staff behavior often stems from work-related stress and unfavorable conditions, emphasizing the need to improve health service delivery settings before blaming providers for disrespectful and abusive behaviors. Specifically, our findings point to the importance of interventional focus on work environment, group dynamics, and the wider health system [42], as well as the need for increasing the intensity of emotional support with greater counseling session frequency [43].

Leadership and management affects facility environments and behavior change interventions targeting clinicians and midwives, as seen from provider and manager concurrence on teamwork and improved supervision. In contrast, resentment, anger, discontent and mistrust - if unaddressed - may result in work-related frustration transferred to patients, resulting in disrespectful and abusive behaviors to clients. This may be further aggravated by poor remuneration and unsupportive supervision structures [44, 45]. Our findings show that dissatisfaction with unresponsive management related to perceptions of fair job promotion can lead to de-motivated health workers. At the same time, unclear guidance on managing human resources in a devolved health system affected provider training and delayed salaries [26, 46]. Resulting provider dissatisfaction with management structures may have contributed to the provider strikes during the intervention period. The need for a more supportive work environment and prevailing policy context is central to provider performance generally and specifically for promoting RMC.

### Limitations

Varied results for composite scores and individual measures of changes in provider knowledge, attitudes, and behaviors illustrate the challenge of measuring the multi-dimensional RMC-promotion processes and outcomes. Factor analyses of measures indicate high reliability of composite scales capturing changes in almost every element, although breakdown analysis of individual items reveals fewer statistically significant improvements over the intervention period. For example, the composite score in management shows improvements following *Heshima* implementation; however, we saw no change (plausibly due to insufficient sample size) in the majority of its individual components. The intervention effect stems primarily from changes in managers’ decision-making behaviors for providers’ needs, addressing provider and client concerns and rights, and treating both with respect, dignity and kindness. This may be due to *Heshima*’s differential influence on specific elements or a function of the loss of variance in composite scores. As such, using composite scores to evaluate changes from baseline to endline ought to be interpreted carefully due to delivery size, facility size, ratio of staff to deliveries, type of facility. Given that this study is one of the first globally to develop these composite measures for RMC, further testing and validation is needed in other settings.

Another limitation stems from this study’s low sample size and lack of a comparison group. A further challenge was the evolving policy context during the project, coupled with provider strikes, which may have affected intervention implementation and efficacy. Devolution of administrative power from the national to county level involved reassignment of districts and prior geographic areas into distinct counties, followed by a progressively phased formation of county health teams – all of which were contemporaneous with *Heshima* implementation and affected provider work environments. The study was unable to follow a specific cohort of providers over time, to capture their individual trajectories (qualitatively or quantitatively), preventing causal claims about effectiveness. Future studies should focus on longitudinal and panel designs to better assess the efficacy and effectiveness of multi-component interventions like *Heshima*.

Despite these limitations, the mixed method implementation research approach presents a set of broad findings that can further be assessed in Kenya and other contexts, and considers provider perspectives over time. It is the first of its kind to try to gather provider perspectives of a multi-component intervention for understanding and addressing women’s mistreatment during childbirth. Though sustaining behavior changes after interventions like *Heshima* is difficult given the complex affective factors, it is possible when accompanied by advocacy, leadership, and partnership with ministries of health, committed partners, and regulatory and professional bodies invested in scaling up of RMC as an integral part of MNH services.

## Conclusions

Behavior change interventions focusing on providers are central to promoting RMC. This study unveils how an intervention package such as *Heshima* affects provider attitudes and practices, is possible to implement, influences RMC promotion, and provides lessons for scale up. Provider working relationships and environment not only impact their own emotional health (burnout), but also can drive poor provider-client interactions and affect women’s care. Factors influencing performance, management, and decision-making remain a challenge for mitigating mistreatment. We must recognize the health providers’ challenging work environments and learn how to support them, daily, for achieving RMC.

## Additional file

Additional file 1: Table A. Individual composite score items assessing knowledge and attitudes of providers participating in baseline (2012) and end line (2014) surveys of the *Heshima* project in 13 facilities in Kenya, *N* = 142. (DOCX 21 kb)

## Abbreviations

D&A: Di srespect and abuse
FIDA: Federation of Women Lawyers in Kenya
HIV: Human immunodeficiency virus
IDIs: In-depth interviews
NNAK: National nurses association Kenya
QITs: Quality improvement teams
RMC: Respectful maternity care
SAS: Statistical analysis system
SD: Standard deviation
URC: University research company.
USAID: United States Agency for International Development
VCAT: Values clarification and attitude transformation
