# Supplementary material for: Exploring provider perspectives on respectful maternity care in Kenya: “Work with what you have”
Source: Reprod Health. 2017 Aug 22;14:99. doi: 10.1186/s12978-017-0364-8 (PMC5567891; doi:10.1186/s12978-017-0364-8)
Supplement: Additional file 1: Table A. — Individual composite score items assessing knowledge and attitudes of providers participating in baseline (2012) and end line (2014) surveys of the Heshima project in 13 facilities in Kenya, N = 142. (DOCX 21 kb) [file 12978_2017_364_MOESM1_ESM.docx]

| **Supplementary Table A: Individual composite score items assessing knowledge and attitudes of providers participating in baseline (2012) and end line (2014) surveys of the *Heshima* project in 13 facilities in Kenya, N = 142** | | | |
| --- | --- | --- | --- |
| **Components of assessment** | **Baseline (n = 67)**  **% (n)** | **Endline (n = 75) % (n)** | **p - value** |
| **Client Rights (14 items)** |  |  |  |
| Informing/orienting the clients of her where about in the facility/ ward/unit | 41.8 (28) | 56.5 (39) | 0.086 |
| Explaining clients when and how procedures will be performed and the out comes | 38.8 (26) | 63.2 (43) | 0.005 |
| Obtaining consent for all procedures to be done | 50.8 (34) | 65.2 (45) | 0.087 |
| Informing client of danger signs during labour and delivery and after birth | 29.9 (20) | 24.6 (17) | 0.495 |
| Informing her of the labour progress and expected possible duration of labour | 32.8 (22) | 43.5 (30) | 0.202 |
| Allowing her to choose her birthing position | 27.3 (18) | 18.8 (13) | 0.244 |
| Allowing her to choose a birth partner/companion during labour and delivery | 17.9 (12) | 24.6 (17) | 0.338 |
| Respecting her privacy | 62.7 (42) | 81.2 (56) | 0.016 |
| Keeping her information confidential | 37.3 (25) | 65.2 (45) | 0.001 |
| Ensuring privacy and confidentiality at all time while attending to client during labour and delivery | 40.3 (27) | 52.2 (36) | 0.165 |
| Respecting her as an individual with her own rights despite her background | 25.4 (17) | 44.9 (31) | 0.017 |
| Ensuring that the procedures are promptly done within the required guidelines | 29.9(20) | 37.7 (26) | 0.335 |
| Ensuring that the mothers labour is monitored using a partograph | 25.4 (17) | 37.7 (26) | 0.123 |
| Taking client to theatre for caesarean section when is not necessary | 3.0 (2) | 7.3 (5) | 0.261 |
| **Emotional Health (22 items)** |  |  |  |
| Not emotionally drained from my work | 40.0 (26) | 44.3 (27) | 0.628 |
| Not used up at the end of the workday. | 36.9 (24) | 32.2 (19) | 0.581 |
| Not fatigued or tired when they get up in the morning and have to face another day on the job. | 47.7 (31) | 40.3 (25) | 0.403 |
| Easily understand how their patients feel about things. | 93.6 (58) | 90.3 (56) | 0.510 |
| Do not treat any patients as if they were impersonal objects | 83.9 (52) | 95.2 (59) | 0.040 |
| Working with people all day is not really a strain for me. | 84.4 (54) | 78.7 (48) | 0.412 |
| Deal very effectively with the problems of their patients. | 90.8 (59) | 88.7 (55) | 0.702 |
| Not burned-out from my work. | 43.8 (28) | 33.9 (21) | 0.255 |
| Positively influence other people's lives. | 87.5 (56) | 83.9 (52) | 0.561 |
| Have not become more callous/hardened toward people since I took this job. | 68.3 (41) | 76.3 (45) | 0.334 |
| The job is not hardening them emotionally. | 72.6 (45) | 64.4 (38) | 0.333 |
| Very energetic. | 82.8 (53) | 68.3 (41) | 0.060 |
| Not frustrated by my job. | 69.7 (46) | 52.5 (32) | 0.046 |
| I do not feel I'm working too hard on my job. | 24.2 (16) | 18.6 (11) | 0.448 |
| Really care what happens to all patients. | 98.5 (65) | 95.2 (59) | 0.354 |
| Working directly with people does not put too much stress on me. | 83.1 (54) | 75.4 (46) | 0.288 |
| Easily create a relaxed atmosphere with their patients. | 90.9 (60) | 90.3 (56) | 0.909 |
| Accomplish many worthwhile things in this job. | 92.1 (58) | 86.7 (52) | 0.331 |
| **E**xhilarated after working closely with their patients. | 63.8 (37) | 66.7 (38) | 0.746 |
| Do not feel like they are at the end of my rope. | 87.9 (58) | 73.8 (45) | 0.043 |
| Deal with emotional problems very calmly. | 89.2 (58) | 78.7 (48) | 0.106 |
| Patients do not blame them for any of their problems. | 75.8 (50) | 70.0 (42) | 0.467 |
| **Client Centred Care (7 items)** |  |  |  |
| I feel that it is always necessary to obtain consent from clients when conducting a vaginal examination | 18.8 (12) | 86.4 (51) | <.0001 |
| If a woman’s uterus rapture during labour and delivery and it becomes necessary for the doctor to perform hysterectomy in order to save her life. It is always necessary to inform her of this unplanned procedure before she is discharged home. | 19.4 (12) | 86.4 (51) | <.0001 |
| On admission mothers, should be allowed to select the provider/s of their choice during labour and delivery. | 59.7 (37) | 36.8 (21) | 0.0128 |
| Mothers who are unable to pay for maternity services should not be detained in the facility to avoid losing the much-needed revenue | 17.5 (11) | 87.7 (50) | <.0001 |
| Treating mothers with care and respect during child birth does make clients come back to this facility | 20.6 (13) | 84.8 (50) | <.0001 |
| During labor and delivery not being harsh to the mothers ensures that they cooperated with you during procedures | 25.4 (16) | 89.8 (53) | <.0001 |
| Sharing of beds in this facility is generally not acceptable to the mothers during labour and delivery | 22.2 (14) | 94.6 (53) | <.0001 |
| **HIV CARE (7 items)** |  |  |  |
| I do not try as much as possible to avoid conducting vaginal exam for the HIV positive clients in the | 46.0 (29) | 68.3 (41) | 0.013 |
| I am comfortable conducting a delivery for a client who is HIV positive | 15.9 (10) | 66.7 (40) | <.0001 |
| I am comfortable repairing a tear or an episiotomy for a HIV positive client | 24.2 (15) | 66.7 (40) | <.0001 |
| I do not feel that during labour and delivery the HIV positive client should be isolated from the rest | 12.7 (8) | 89.8 (53) | <.0001 |
| I am comfortable caring for mother who has HIV soon after delivery | 17.7 (11) | 66.1 (39) | <.0001 |
| I am comfortable nursing babies born of mother who are HIV positive | 17.7 (11) | 71.2 (42) | <.0001 |
| Recording client’s positive HIV status on the client’s card/mother baby booklet make some of the clients uncomfortable | 23.8 (15) | 54.2 (32) | 0.001 |
| **Management (14 items)** |  |  |  |
| Job decisions are made by my manager in an unbiased manner. | 45.9 (28) | 49.2 (30) | 0.717 |
| My manager makes sure that all staff concerns are heard before job decisions are made. | 38.1 (24) | 41.9 (26) | 0.661 |
| To make job decisions, my manager collects accurate and complete information. | 30.2 (199) | 45.2 (28) | 0.083 |
| My manager clarifies decisions and provides additional information when requested by staff. | 17.7 (11) | 53.2 (33) | <.0001 |
| All job decisions are applied consistently across all affected staff. | 35.5 (22) | 40.3 (25) | 0.579 |
| Staff are allowed to challenge or appeal job decisions made by my manager. | 46.0 (29) | 50.0 (31) | 0.657 |
| My manager offers adequate justification for decisions made about my job. | 36.5 (23) | 50.0 (31) | 0.128 |
| When making decisions, … |  |  |  |
| …my manager treats me with kindness and consideration. | 20.3 (13) | 66.1 (41) | <.0001 |
| …my manager treats me with respect and dignity. | 20.3 (13) | 67.7 (42) | <.0001 |
| …my manager is sensitive to my personal needs. | 37.5 (24) | 61.3 (38) | 0.008 |
| …my manager deals with me in a truthful manner. | 25.4 (16) | 61.3 (38) | <.0001 |
| …my manager shows concern for my rights as an employee. | 31.3 (20) | 60.7 (37) | 0.001 |
| …my manager discusses the implications of the decisions with me. | 31.3 (20) | 50.0 (31) | 0.032 |
| …my manager offers explanations that make sense to me. | 23.4 (15) | 56.5 (35) | 0.0002 |
| **Job Fairness (5 items)** |  |  |  |
| My work schedule is fair. | 34.4 (21) | 64.5 (40) | 0.001 |
| I think that my level of pay is fair. | 69.4 (43) | 11.3 (7) | <.0001 |
| I consider my workload to be quite fair | 68.3 (43) | 21.0 (13) | <.0001 |
| Overall the rewards I receive here are quite fair. | 77.4 (48) | 16.1 (10) | <.0001 |
| I feel that my job responsibilities are fair. | 45.2 (28) | 56.5 (35) | 0.209 |
| **Supervision (5 items)** |  |  |  |
| I think this is a fair supervision system. | 23.1 (15) | 65.0 (39) | <.0001 |
| I feel good about this supervision system. | 24.2 (16) | 68.3 (41) | <.0001 |
| I am satisfied with this supervision system. | 26.2 (17) | 46.7 (28) | 0.017 |
| The feedback I receive is fair | 21.9 (14) | 49.1 (27) | 0.002 |
| I think my supervisor are knowledgeable for effective supervision | 13.6 (9) | 63.3 (38) | <.0001 |
| **Promotion (5 items)** |  |  |  |
| *I think this is a fair promotion system.* | 41.7 (25) | 54.4 (31) | 0.169 |
| I feel good about this promotion system. | 38.3 (23) | 52.6 (30) | 0.120 |
| I am satisfied with this promotion system. | 38.3 (23) | 51.7 (30) | 0.144 |
| The promotion opportunities I have are fair. | 38.3 (23) | 50.0 (29) | 0.202 |
| Compared to other people doing similar work, my opportunities for promotion are fair. | 41.7 (25) | 45.6 (26) | 0.667 |
| **Health System (9 items)** |  |  |  |
| I think this is a fair system. | 34.9 (22) | 32.1 (18) | 0.749 |
| I feel good about this system. | 36.5 (23) | 30.9 (17) | 0.522 |
| I am satisfied with this system. | 44.4 (28) | 25.0 (14) | 0.027 |
| This system provides fair training opportunities. | 50.0 (31) | 32.1 (18) | 0.049 |
| The outcome of this system is that I get the training I deserve. | 46.0 (29) | 28.6 (16) | 0.050 |
| I am adequately trained for all the tasks I perform. | 54.8 (34) | 30.9 (17) | 0.009 |
| Most of the training I have received has improved or changed how I practice. | 8.1 (5) | 69.6 (39) | <.0001 |
| The training I have received in general has been of a high quality. | 15.9 (10) | 73.2 (41) | <.0001 |
| The facility management has offered me an opportunity to practice post in-service training | 28.3 (17) | 52.7 (29) | 0.008 |
| **Work Environment (13 items)** |  |  |  |
| Enough staff to provide quality patient care | 24.2 (16) | 22.6 (14) | 0.825 |
| Enough staff to get the work done | 25.0 (16) | 36.7 (22) | 0.159 |
| Opportunity to work on a highly-specialized patient care unit | 44.6 (29) | 46.6 (27) | 0.830 |
| Adequate support services allow health workers to spend time with patients | 53.0 (35) | 60.7 (37) | 0.386 |
| Freedom to make important patient care and work decisions | 74.2 (49) | 72.6 (45) | 0.832 |
| Patient care assignments that support continuity of care, i.e., the same health workers care for the patient from one day to next | 63.1 (41) | 62..9 (39) | 0.984 |
| Health professionals control their own practice | 66.7 (44) | 64.5 (40) | 0.798 |
| Adequate pre-service education for my current position | 58.5 (38) | 67.7 (42) | 0.279 |
| Adequate clinical practical opportunities during pre-service training | 63.1 (41) | 78.7 (48) | 0.055 |
| Adequate opportunities for professional development and career | 50.8 (33) | 55.7 (34) | 0.577 |
| Adequate clinical supervision in this service | 70.8 (46) | 68.3 (41) | 0.767 |
| Consistent availability of supplies and medications to perform my duties | 45.5 (30) | 57.4 (35) | 0.179 |
| Functioning equipment and infrastructure to perform my duties | 53.0 (35) | 62.3 (38) | 0.291 |
| **Working Relationships (14 items)** |  |  |  |
| Enough time and opportunity to discuss patient care problems with other staff. | 56.9 (37) | 50.0 (31) | 0.434 |
| A manager who is provides support supervision and leadership | 84.9 (56) | 82.3 (51) | 0.693 |
| A manager who backs up the staff in decision-making and conflict resolution even if the conflict is within cadre, below or with a more qualified member of staff | 75.8 (50) | 83.9 (52) | 0.254 |
| Hospital/clinic managers support and value health workers | 75.8 (50) | 74.2 (46) | 0.838 |
| Doctors, nurses and other health workers have good working relationships | 80.30 (53) | 79.0 (49) | 0.858 |
| Medical Officers have good working relationships with Clinical Officers | 85.9 (55) | 81.7 (49) | 0.518 |
| Nurses have good working relationships with Clinical Officers | 87.9 (58) | 85.5 (53) | 0.690 |
| Medical officers have good working relationships with Nurse midwives | 84.6 (55) | 83.6 (51) | 0.877 |
| Obstetricians have good working relationship with midwives | 88.9 (56) | 82.5 (47) | 0.313 |
| Enrolled nurses have good relationships with registered nurses | 84.9 (56) | 98.4 (60) | 0.007 |
| Nurses have good relationships with Medical Officers /interns | 85.7 (48) | 84.5 (49) | 0.854 |
| Nurses have good relationships with doctors | 84.4 (54) | 88.5 (54) | 0.499 |
| Collaboration (joint practice) between different cadres of health workers | 81.5 (53) | 88.5 (54) | 0.273 |
| A lot of team work between different cadres of health workers | 81.8 (54) | 80.3 (49) | 0.830 |
